# Development of *Synechocystis* sp. PCC 6803 as a Phototrophic Cell Factory 

**DOI:** 10.3390/md11082894

**Published:** 2013-08-13

**Authors:** Yi Yu, Le You, Dianyi Liu, Whitney Hollinshead, Yinjie J. Tang, Fuzhong Zhang

**Affiliations:** 1 Key Laboratory of Combinatory Biosynthesis and Drug Discovery (Ministry of Education), School of Pharmaceutical Sciences, Wuhan University, 185 East Lake Road, Wuhan 430071, China; E-Mail: yuyi@seas.wustl.edu; 2 Department of Energy, Environmental and Chemical Engineering; Washington University, St. Louis, MO 63130, USA; E-Mails: leyou@wustl.edu (L.Y.); whollinshead@wustl.edu (W.H.); 3 Division of Biology & Biomedical Sciences, Washington University, St. Louis, MO 63130, USA; E-Mail: dianyi.eden@gmail.com

**Keywords:** algae, biofuel, bioprocess scale-up, metabolism, systems biology

## Abstract

Cyanobacteria (blue-green algae) play profound roles in ecology and biogeochemistry. One model cyanobacterial species is the unicellular cyanobacterium *Synechocystis* sp. PCC 6803. This species is highly amenable to genetic modification. Its genome has been sequenced and many systems biology and molecular biology tools are available to study this bacterium. Recently, researchers have put significant efforts into understanding and engineering this bacterium to produce chemicals and biofuels from sunlight and CO_2_. To demonstrate our perspective on the application of this cyanobacterium as a photosynthesis-based chassis, we summarize the recent research on *Synechocystis* 6803 by focusing on five topics: rate-limiting factors for cell cultivation; molecular tools for genetic modifications; high-throughput system biology for genome wide analysis; metabolic modeling for physiological prediction and rational metabolic engineering; and applications in producing diverse chemicals. We also discuss the particular challenges for systems analysis and engineering applications of this microorganism, including precise characterization of versatile cell metabolism, improvement of product rates and titers, bioprocess scale-up, and product recovery. Although much progress has been achieved in the development of *Synechocystis* 6803 as a phototrophic cell factory, the biotechnology for “Compounds from *Synechocystis*” is still significantly lagging behind those for heterotrophic microbes (e.g., *Escherichia coli*).

## 1. Introduction

Cyanobacteria grow in many different regions throughout the world. They have contributed significantly to both the food chains and the atmospheric oxygen levels, promoting ecological biodiversity. Worldwide, cyanobacteria convert solar energy into biomass-based chemical energy at a rate of ~450 Terawatts, which is >25 times higher than the total power used by humans [1]. Their powerful phototrophic metabolism has sparked research into utilization of cyanobacteria for generating renewable biofuels and chemicals using sunlight and the greenhouse gas CO_2_ [2,3]. Among all cyanobacterial species, *Synechocystis* sp. PCC 6803 (hereafter *Synechocystis* 6803) is one of the most extensively studied species since it was initially isolated from a freshwater lake in 1968. The entire genome, including four endogenous plasmids, was sequenced, and over 3000 genes have been annotated to date [4,5]. *Synechocystis* 6803 demonstrates versatile carbon metabolisms, growing under photoautotrophic, mixotrophic and heterotrophic conditions [6]. Additionally, biochemical similarities between the plant chloroplasts and *Synechocystis* 6803 make the latter an ideal system for studying the molecular mechanisms underlying stress responses and stress adaptation in higher plants [7]. More importantly, this species is naturally competent (homologous recombination at high frequency) [8]. The recent developments in synthetic biology have provided plenty of molecular biology tools to engineer *Synechocystis* 6803 as a photosynthetic host for the production of diverse types of chemicals. In this review paper, we mainly focus on advances in the application of *Synechocystis* 6803 for biosynthesis as well as the development of tools for rational genetic modification and bioprocess scale-up. This overview of *Synechocystis* 6803 presents our perspectives on the challenges in cyanobacterial research related to functional characterization, rational metabolic engineering, and bioprocess engineering. 

## 2. Influential Factors for *Synechocystis* 6803 Cultivation

*Synechocystis* 6803 bio-productions require robust biomass growth. Optimal cyanobacterial cultivation depends on both nutrient availability (CO_2_, nitrogen, and phosphorus) and cultivation conditions (light irradiance, temperature, pH,* etc.*) [9]. Many studies have been performed to reveal *Synechocystis* physiology under nutrient “replete”* versus* “deplete” conditions, as well as its metabolic responses to environmental factors. 

### 2.1. CO_2_ Fixation

*Synechocystis* 6803 is able to fulfill oxygenic photosynthesis and CO_2_ fixation. Ribulose-1,5-bisphosphate carboxylase (RuBisCO) catalyzes the first step of the Calvin Cycle for CO_2_ fixation. However, this enzyme is sensitive to O_2_. To solve this problem, *Synechocystis* has evolved a unique organelle, carboxysome, which encapsulates RuBisCO and concentrates inorganic carbon for biomass growth. In detail, the carboxysome membrane is rather impermeable to O_2_, but HCO− 3 in the cytosol is able to diffuse into the carboxysome via active transport [10]. Such subcellular localization of RuBisCO provides a useful barrier to O_2_ diffusion so that *Synechocystis* photorespiration (oxidation of Ribulose-1,5-bisphosphate by O_2_ that competes with carbon fixation) can be maintained at a very low level during autotrophic growth (<1% of total carbon fixation) [11]. 

Under CO_2_ limitation conditions, studies using the shotgun Liquid Chromatography-Mass Spectrometry (LC-MS/MS) method have revealed significant proteomic changes mediated by multiple transcriptional regulators in *Synechocystis* 6803, including proteins participating in inorganic carbon fixation, nitrogen transport and assimilation, as well as in the protection of the photosynthetic machinery from excess light [12]. Low inorganic carbonate concentration also activates the *Synechocystis* 6803 CO_2_-concentrating mechanism [13] via the induction of a highly efficient bicarbonate transporter (bicarbonate-binding protein CmpA) [14]. Therefore, *Synechocystis* 6803 has the capability to grow under very low CO_2_ concentrations [12,15]. 

### 2.2. Organic Carbon Utilization

*Synechocystis* 6803 can grow either photoautotrophically via the Calvin Cycle (maximum doubling time 7~10 h under optimal light conditions) or photoheterotrophically on glucose via the glycolysis pathway and the oxidative pentose phosphate pathway (doubling time ~3.5 days). Some sub-strains of *Synechocystis* 6803 cannot utilize glucose as the main carbon source [16,17,18,19]. Glucose-tolerant *Synechocystis* 6803 sub-strains will not grow on glucose under complete darkness unless given a daily pulse of white light [20]. Moreover, our recent study indicated that glucose tolerant *Synechocystis* 6803 may co-metabolize different organic acids (including pyruvate, acetate, and succinate) when inorganic carbon (CO_2_ or HCO− 3) is insufficient in the medium [21]. Although the use of organic acids as cyanobacterial feedstock is not cost-effective for scale-up biomass production, the organic substrate utilization capability may provide insight into the evolutionary transition from autotrophic to heterotrophic metabolisms in early life. 

### 2.3. Light Harvesting

Previous studies have revealed that under sufficient irradiance and CO_2_ concentrations, RuBisCO is not the rate-limiting factor for *Synechocystis* 6803 photosynthetic growth. Rather, autotrophic growth is constrained by phosphoglycerate reduction due to ATP/NADPH limitation from photo-reactions [22]. Cyanobacteria cannot absorb all incoming sunlight due to light reflection, dissipation, shading effect, and the limited absorption spectrum (blue and red) of the photosynthetic antenna. Furthermore, photons absorbed by antennae cannot be fully used for energy conversion. Therefore, antenna truncation has been proposed to increase the efficiency of the light harvesting system [23]. Unfortunately, antenna modification has not produced any advantages in biomass production in *Synechocystis* 6803 [24]. With electron microscopy and hyper spectral confocal fluorescence microscopy, Collins* et al.* revealed that phycobilisome truncation mutants exhibit decreased concentrations of both photosystem I and photosystem II [25]. Currently, light harvesting is still the rate-limiting step for high-efficiency *Synechocystis* biomass growth.

### 2.4. Nitrogen and Phosphorus Uptake

Most cyanobacteria are able to use nitrate and ammonium as nitrogen sources [26,27]. *Synechocystis* 6803 cannot fix N_2_ naturally (absence of nitrogenase), but it is able to store ammonium nitrogen inside of the cell by producing cyanophycin, a polypeptide containing multiple arginine and aspartate residues. This polymer can be degraded by a hydrolytic enzyme cyanophycinase under nitrogen limitation condition [28]. Such nitrogen-storage capability of *Synechocystis* 6803 can be potentially used for removal of nitrogen from wastewater, and the resulting cyanophycin can be used as a bio-fertilizer. On the other hand, Krasikov and coworkers monitored the transcriptome of *Synechocystis* 6803 after nitrogen starvation [26], and observed three nitrogen starvation responses: (1) nitrogen assimilation is up-regulated; (2) cells exhibit chlorosis (the pigment degradation process of cyanobacteria) with a transcriptional repression of the phycobilisome genes; and (3) cells stop most of their enzymatic activities. In addition, photosynthetic carbon fixation is limited under nitrogen depletion conditions (*i.e.*, RuBisCO encoding genes are strongly down-regulated) [29].

*Synechocystis* 6803 is capable of accumulating inorganic polyphosphate (polyP) inside the cell. Deletion of its phosphate regulator genes (*phoU* and *sphU*) significantly increases the intracellular polyP concentration; thus, such mutants can be used for Pi removal from wastewater [30]. Pi starvation enhances cyanobacterial phosphorus uptake. However, phosphorus deprivation harms photosynthetic machinery and slows down both energy and nucleotide sugar generation [31]. In general, both Pi and N depletion are coupled with the down-regulation of photosynthesis, and trigger the metabolic responses to maintain homeostasis. Chlorosis is observed under nitrogen or phosphorus deprivation [32]. Therefore, N and Pi supplements are crucial for *Synechocystis* 6803 cultivation, which accounts for the main cost in the scale-up of *Synechocystis* cultivation. 

### 2.5. Cultivation Conditions

*Synechocystis* 6803 is commonly cultivated at 30 °C, using BG11 medium (pH = 7~8). Cells can maintain their growth in oxic conditions (or photo-bioreactors) and alkaline medium (pH = 10). Elemental analysis of nutrient requirements showed the *Synechocystis* 6803 dry biomass formula is CH_1.62_O_0.40_N_0.22_P_0.01_. In ideal photo-bioreactors, *Synechocystis* 6803 achieves a specific growth rate of 1.7~2.5 day^−1^ and a nitrate uptake rate of 0.46 g N/g dry cell weight day^−1^ [9]. Moreover, *Synechocystis* metabolism has unique responses to environmental stresses (e.g., cold-stress, hyperosmotic stress and salt stress). In unfavorable environments, *Synechocystis* 6803 shows activation of alternate pathways for the acquisition of carbon and nitrogen (e.g., breakdown of cyanophycin into arginine and aspartic acid) and for the reduction of photosynthesis efficiencies. Under such conditions, the specific growth rate could be less than 1 day^−1^. Thereby, environmental conditions are important considerations for algal bioprocess scale-up, especially for open pond systems.

In summary, when compared to *Escherichia coli* or *Saccharomyces cerevisiae* (specific growth rates >10 day^−1^), one of the major limitations in large-scale cultivation of *Synechocystis* 6803 is the slow growth. Although optimization of cultivation conditions in photoreactors could resolve this problem to some extent, high cost associated with photoreactor manufacturing and operation limits the large-scale cyanobacterial production. Alternatively, there might be an answer with the development of synthetic biology and systems biology. Hopefully, bottlenecks for cyanobacterial bioprocess will be solved via systems metabolic engineering in the future.

## 3. Genetic and Molecular Tools

To produce chemicals in a heterologous host, it is essential to manipulate its genetic codes and control gene expression levels so that carbon can flow through the desired pathways. Synthetic biology has advanced the development of numerous genetic and molecular biology tools for model microbial hosts (such as *E. coli and S. cerevisiae*). Similar tools are being developed for *Synechocystis* 6803, allowing for genetic manipulation at transcriptional, translational, and posttranslational levels.

### 3.1. Plasmid Vectors

Expression of heterologous genes requires introduction of DNAs to the host, either on plasmids or in the chromosome. The two types of plasmid vectors that are typically used are integrative and replicative plasmids. Integrative plasmids integrate a gene into the genomic DNA of *Synechocystis* 6803 by homologous recombination [33]. Such plasmids do not survive long inside the expression host and are designed to insert a desired gene into specific sites of the genomic DNAs. The most representative integrative vector is pTCP2031V, which integrates the target gene to the *slr2031* site of the *Synechocystis* chromosome [34]. Besides *slr2031*, other neutral sites, such as the *psbA1* gene locus, were also used to construct high-efficiency integrative vectors [35]. In contrast, replicative plasmids allow fast introduction of heterologous genes into the host. RSF1010 is an *E. coli*-derived shuttle vector and is used extensively in *Synechocystis* 6803 as a replicative plasmid [2,36]. Other shuttle vectors that have been developed in *Synechocystis* 6803 include pFC1 [37], pFF11 [38], and pFCLV7 [39]. However, replicative vectors are genetically less stable, and antibiotic pressure is usually required for continuous proliferation of the vector in the host [40]. 

### 3.2. Transformation and Segregation

*Synechocystis* 6803 during the exponential growth phase has high transformation efficiencies through natural transformation, ultrasonic transformation, or electroporation [8]. For natural transformation, a pretreatment with EDTA for two days increased efficiency by 23%. Transformation efficiencies for circular DNA have been shown to be ~30% higher than linear DNA [8]. Successful transformation can be increased by two orders of magnitude via deletion of *sll1354* (the gene encoding the exonuclease RecJ) [41]. However, it should be noted that among sub-strains that bear the common name *Synechocystis* 6803, the Kazusa strain is non-competent for transformation [18,42]. Because *Synechocystis* 6803 contains more than one genome copy per cell, only one chromosome is involved in the recombination event once the integrative vector is introduced [6]. To obtain a genetically homogeneous recombinant strain, segregation with selection pressure is necessary, by continuously streaking the colonies onto plates containing increasing level of antibiotics [6]. Therefore, the choice of the integration site in *Synechocystis* 6803 is very important because complete segregation is impossible if the inactivation of the gene locus confers significant disadvantages to the host growth.

### 3.3. Transcriptional Control Tools

Gene expression can be controlled by proper promoters. Several *Synechocystis* 6803 native promoters are used to overexpress heterologous proteins. P_rbcLS_ and P_psbA2_ are strong native promoters involved in the expression of RuBisCO and photosystem II, respectively [2]. They have been used to control *pdc* and *adh* genes for ethanol synthesis [43], or to overexpress the fatty acid pathway(*accBCDA*/*tesA*/*fatB2* genes) [44]. Some native promoters have the ability to control gene expression according to environmental signals (e.g., light cycles/intensity, nitrogen-stress, salt-stress, and temperature), allowing expression levels of the controlled genes to respond to environmental changes. For example, promoters associated with genes* clpP*, *slr1634*, and *rbpP* showed distinctive circadian patterns [45], which could express heterologous genes with a diurnal rhythm. To express a pathway with multiple enzymes, inducible promoters that reduce stress from early gene expression are advantageous. The *Synechocystis* 6803 nickel-sensing system, NrsR and NrsS (encoded by *nrsR* and *nrsS*), detects extracellular nickel concentrations and triggers gene expression from the *nrsBACD* promoter. Placing heterologous genes under the control of P_nrsBACD_ would allow the gene expression to be nickel-inducible [46]. Recently, Huang* et al.* characterized a series of inducible promoters commonly used in *E. coli* and found most of them lost their inducibility when used in *Synechocystis* 6803 [47]. For *Synechocystis*, P_trc_ led to high expression levels in the absence of its inducer (leaky), while P_lac_, P_tet_, and P_R_ have low expression levels even with high inducer concentrations. It is still not clear why these promoters behaved differently in *E. coli* and *Synechocystis* 6803. Several factors could cause such differences including the specificity and concentration of sigma factors, inducer membrane permeability, and unknown regulators that may interact with these promoters. Currently, *Synechocystis* 6803 still lacks non-leaky, inducible promoters with a large inducible range. 

As a part of the RNA polymerase holo-enzyme complex, sigma factors play central roles in transcription initiation. *Synechocystis* 6803 has evolved specialized sigma factors that can be divided in three groups: sigma factors with consistent expression throughout growth, sigma factors that respond to specific environmental conditions, and sigma factors related to motility [48]. For example, engineering of sigma factor SigE increased the level of many sugar catabolic enzymes in *Synechocystis* 6803 [49]. Furthermore, transcription factors (both repressors and activators) together with their cognate promoters can be potentially used to create biosensors for intracellular signals in *Synechocystis* 6803 [50,51]. 

### 3.4. Translational Control Tools

Gene expression can be controlled at the level of translation. The ribosomal binding site (RBS) is an important genetic element involved in mRNA translation initiation. In prokaryotes, there is a correlation between the RBS sequence and the protein level of its downstream gene [52]. Prediction and optimization of the RBS strength could be guided by thermodynamic models (to predict interaction of mRNA with synthetic RBS) [33,53]. Other post-transcriptional control tools include RNA processing and RNA silencing and antisense RNAs (asRNAs), which play crucial roles in gene translational regulation in eukaryotic cells [54]. AsRNAs have been extensively found in prokaryotes [55], including *Synechocystis* 6803 [56]. For example, IsrR is a transcript from the noncoding strand of *isiA*, which is an iron stress-induced gene. The IsrR/*isiA* mRNA duplex is degraded in normal conditions. Under continuous iron deficiency, transcription of *isiA* will exceed that of IsrR and accumulated IsiA influences photosynthesis apparatus reorganization. Thus, IsrR acts as a photosynthesis-related RNA silencer in *Synechocystis* 6803. In other studies, several asRNAs are found to suppress the translation of genes involved in *Synechocystis* photosystem II. So far, there are ~73 asRNAs in *Synechocystis* 6803 and ~10% of annotated genes are associated with asRNA [55,57]. Moreover, clustered regularly interspaced short palindromic repeats (CRISPR) is a type of small RNAs-based foreign nucleic acid silencing system analogous to RNAi in eukaryotic organisms [58], which can be used for RNA-guided translation engineering. There are several CRISPR-associated genes that have been discovered in *Synechocystis* 6803 [59,60], offering a new tool to control *Synechocystis* gene expression. 

### 3.5. Posttranslational Control Tools

Protein degradation system plays significant roles in cell metabolism via elimination of misfolded and damaged proteins. Degradation tags are short peptide sequences that mark a protein for degradation. They have been used to control protein expression in many microbial systems including *Synechocystis* 6803 [47,61]. For example, engineering the amino acid sequence of the SsrA tag leads to a series of protein degradation tags with various strengths in *Synechocystis* 6803 [62]. Therefore, it is possible to control cellular enzyme levels that affect the flux of metabolic pathways.

In summary, the advances of synthetic biology in *Synechocystis* 6803 are still far behind that in *E. coli.* Many standard biological parts that are widely used in *E. coli* did not work properly in *Synechocystis* 6803 [33]. Taken into account the additional challenges, such as the thylakoid membrane and the multiple-copy chromosomes, it might take extra efforts to develop synthetic biology tools for *Synechocystis* 6803. Due to the importance for genetic engineering, a new set of synthetic biology tools standardized for *Synechocystis* 6803 should be created in the near future.

## 4. High Throughput System Biology for *Synechocystis* 6803

Rational engineering of biosynthetic pathways requires understanding of cell-wide metabolism. High-throughput “omics” (transcriptomics, proteomics, and metabolomics) tools have been applied widely to analyze *Synechocystis* 6803’s dynamic process under various physiological conditions [63].

### 4.1. Transcriptomics

Transcriptomics can provide detailed transcriptional profiles of metabolic pathways under diverse physiological situations. Quantitative reverse-transcript PCR is a traditional method to reveal the metabolic responses in *Synechocystis* 6803. However, high throughput microarray surpasses PCR-based transcriptional analysis. For example, microarray analyses of 163 transcriptome data sets, previously generated in *Synechocystis* 6803, are used to identify coordination and interaction between each cellular process [64]. The study found that many genes associated with photosynthesis, energy metabolism, and translation are commonly regulated together to help cells adapt under stressed conditions [64]. RNA-seq (Transcriptome Sequencing) has offered an accurate and comprehensive view of global cellular responses in *Synechocystis* 6803. This new method has been used to search for potential genes that could improve ethanol tolerance in *Synechocystis* 6803 [65]. Moreover, Yoshikawa* et al.* applied transcriptomics to analyze central cellular metabolism of *Synechocystis* 6803 under different trophic conditions [19]. The results showed that the expression level of most genes related to central metabolism was unchanged under auto- and mixotrophic conditions. However, several key genes involved in the glycolytic and pentose phosphate pathways (such as *gap1*, *gnd* and *rpiA*) exhibited a higher transcriptional level under mixotrophic conditions than autotrophic conditions. 

### 4.2. Proteomics

Proteomics mainly focuses on the determination of protein expression levels, protein-protein interactions, and proteins’ roles in cellular processes. Proteomics can provide functional information on metabolic regulation. It has been used to probe the autotrophic metabolism in *Synechocystis* 6803 under different CO_2_ concentrations [12]. Via shotgun LC-MS/MS and Western blot approaches, *Synechocystis* 6803 proteomic responses to CO_2_ limitation and excess light have been revealed [12]. Moreover, the response of *Synechocystis* 6803 to toxic chemicals (hexane, ethanol, and butanol) has been investigated by iTRAQ-LC-MS/MS [66,67,68]. When treated with these chemicals, the cell induces a large number of proteins involved in photosynthesis, molecular transport, sulfur relay system, cell membrane/envelope modification, heat-shock, and cell mobility, implying that *Synechocystis* 6803 can employ multiple pathways to overcome chemical toxicity. The discovered stress-resistant proteins can be potentially overexpressed in the engineered *Synechocystis* 6803 host to improve their tolerance to the toxic products.

### 4.3. Metabolomics

Metabolomics focuses on low-molecular-weight metabolites. Metabolomics is the consequence of various enzymatic reactions and provides the most straightforward characterization of metabolic responses to genetic modifications or environmental changes. Compared to other omics studies, few metabolomic research studies have been performed in *Synechocystis* 6803. Krall* et al.* have compared three sampling strategies including quenching, filtering, and centrifugation, using a gas chromatography-mass spectrometry (GC-MS)-based metabolomics analysis in *Synechocystis* 6803 [69]. This study found that sampling is very important in metabolomic analyses because many metabolites turn over quickly. Both fast-filtering and centrifugation are better than cold methanol-water quenching for maintaining the integrity of the metabolite pool produced in *Synechocystis* 6803. In a recent study, transcriptomics and metabolomics were integrated to analyze *Synechocystis* 6803 under either autotrophic or mixotrophic conditions [19]. Metabolomics analysis revealed that the oxidative pentose phosphate pathway and glycolysis in cyanobacteria are active under mixotrophic conditions rather than autotrophic conditions. The results also showed that the obtained transcriptomic datasets have low connection with metabolomic datasets, indicating omics data at different levels do not necessarily correlate. This study suggests a crucial need for integrative studies utilizing multiple omics strategies to accurately describe cellular regulations and functions [19].

In summary, omics applications in *Synechocystis* 6803 are still in their infancy. Although omics technologies could provide “big data” on the levels of transcripts, proteins, and metabolites, elucidation of detailed molecular mechanisms still requires large quantities of subsequent* in vivo* and* in vitro* experiments. Ultimately, omics analysis will be used to systematically guide metabolic engineering to improve the efficiency of the engineered *Synchocystis* 6803 cell factory.

## 5. Metabolic Modeling for *Synechocystis* 6803

**Figure 1:**
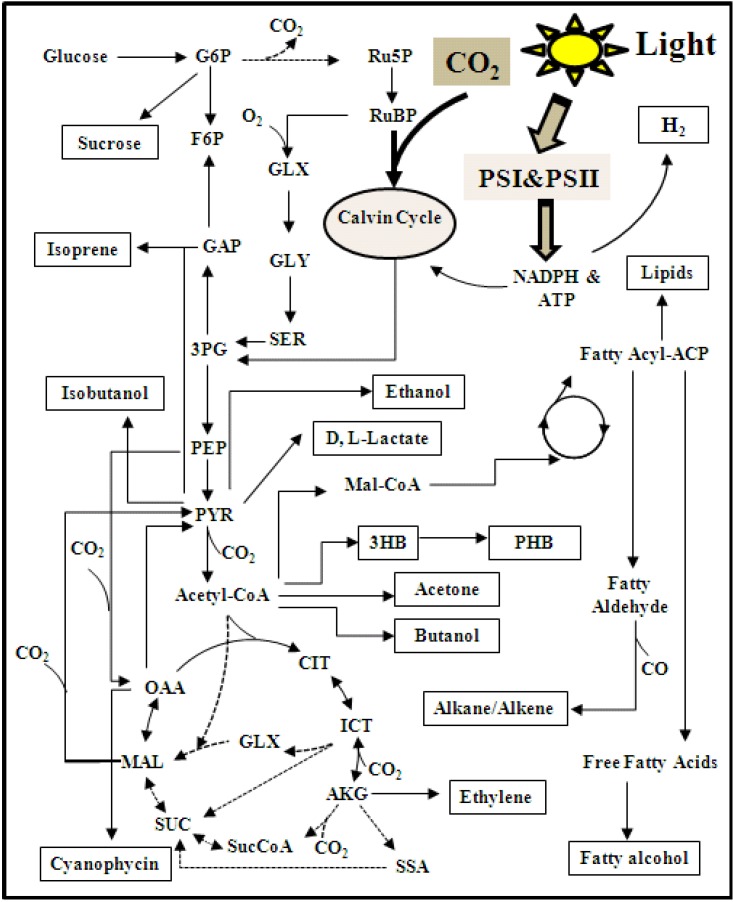
Central metabolic pathways and products from *Synechocystis* 6803. The functions of several pathways (marked as dot-lines) are still not verified. Abbreviation: 3PG: 3-phosphoglycerate; 3HB: 3-hydroxybutyrate; AKG: α-ketoglutarate; CIT: citrate; F6P: fructose 6-phosphate; G6P: glucose 6-phosphate; GAP: glyceraldehyde 3-phosphate; GLX: glyoxylate; GLY: glycine; ICT: isocitrate; MAL: malate; Mal-CoA: Malonyl-CoA; OAA: oxoacetate; PEP: phosphoenolpyruvate; PHB: polyhydroxybutyrate; PSI&PSII: photosystem I & photosystem II; PYR: pyruvate; Ru5P: ribulose-5-phosphate; RuBP: ribulose-1,5-diphosphate; SER: serine; SUC: succinate; SucCoA: succinyl-CoA; SSA: succinic semialdehyde.

Our knowledge regarding *Synechocystis* 6803 metabolism is still not complete (Figure 1). For example, it is well-accepted that *Synechocystis* 6803 has a broken TCA cycle [70]. However, a recent research study has discovered that two new enzymes, which are widely present in cyanobacterial species, can close the TCA cycle [71,72]. Therefore, the function of the TCA cycle in *Synechocystis* 6803 is still under debate. Moreover, the presence of the oxidative pentose phosphate (OPP) pathway under light conditions in *Synechocystis* 6803 is controversial [73,74,75,76]. To provide a clear picture for *Synechocystis* 6803 metabolism, model-based analyses have been coupled with experimental data and bio-informatics to characterize *Synechocystis* physiology.

### 5.1. Flux Balance Analysis (FBA)

FBA can quantitatively characterize the metabolic properties of genome-scale networks [77,78]. Based on the mass balance of all the metabolic intermediates in the metabolic network, a steady-state stoichiometric model is constructed including a set of linear equations for all metabolic reactions. External input and output measurements are employed as constraints. The model is then determined by satisfying a given objective function, e.g., maximization of biomass or a certain product. Shastri and Morgan were the first to reconstruct the metabolic network of *Synechocystis* 6803 using a flux balance approach [79]. The hetero-, auto-, and mixotrophic metabolisms under optimal growth conditions were evaluated and compared. Subsequently, a high-quality genome-scale metabolic network was applied to identify two photosynthetic apparatus and highlight the high photosynthetic robustness in *Synechocystis* 6803 [80]. However, the objective function required in FBA may not always represent the real cell physiology [81]. Besides, due to poorly determined stoichiometric systems, the metabolic model may have multiple solutions that meet the object function equally well [82,83]. Additional constraints are needed for the accurate description of cell suboptimal metabolism [84].

### 5.2. Flux Coupling Finder (FCF)

The FCF framework elucidates the topological and flux connectivity features of genome-scale metabolic networks [85]. Through efficient assessing and comparing of the outcomes of each deletion, FCF identifies the optimal location for equivalent knockouts among multiple targets. FCF investigates the autotrophy, mixotrophy, heterotrophy, and light-activated heterotrophy in *Synechocystis* 6803, and identifies bottlenecks for hydrogen and ethanol production (e.g., cofactor balances and CO_2_ fixation). Integration of FCF with transcriptomic data also reveals new insight into metabolic shifts triggered by the availability of light [86]. Such a framework shows the ability to identify rate-limiting enzymes during strain engineering. 

### 5.3. ^13^C Metabolic Flux Analysis (^13^C-MFA)

^13^C-MFA can provide precise information of intracellular fluxes [87]. This method is based on the isotopic fingerprints of metabolic products under a defined ^13^C-substrate [88]. The labeling information not only highlights the functional pathways and fills the gaps in the genome map [88,89], but also determines absolute carbon fluxes through the metabolic network. ^13^C-MFA has been efficiently applied to analyze photomixotrophic and heterotrophic metabolism [90]. The photoautotrophic metabolism depends on a non-steady-state ^13^C-pulse to capture the isotopic dynamics in free metabolites. Recently, a non-steady state ^13^C pulse method [91] and isotopically non-stationary metabolic flux analysis (INST-MFA) have been developed to investigate the fluxes through photoautotrophic metabolisms [11]. INST-MFA still faces two difficulties. First, isotopomer analysis of low abundant metabolites by GC-MS or LC-MS is technically difficult. Second, the presence of enzyme channeling in the Calvin cycle and glycolysis pathway provides alternative carbon routes in *Synechocystis* 6803 (*i.e.*, channeling may cause more ^13^C-accumulation in the downstream metabolites than their precursors during dynamic labeling experiments) and thus complicates model calculations [11,91].

## 6. Applications of *Synechocystis* 6803 as a Cell Factory

*Synechocystis* 6803 has been used as the host strain for the production of biofuels, commodity chemicals, biomaterials, and health-related compounds (Table 1). 

**Table 1:** Comparing three cyanobacterial species for chemical synthesis (DCW: dry cell weight).

Strains	Chemicals	Genetic modification	Productivity	Growth conditions	References
***Synechocystis*** **6803**	Ethanol	*pdc* and *slr1192*; Δ*phaAB*	5.50 g/L	photoautotrophic; Sparging with 5% CO_2_-air	[92]
		Promoter *rbc*			
	Fatty acids	*tesA*, *tesA137* (codon optimized), *accBCDA*, *fatB1*, *fatB1*, *fatB2*; Δ*phaAB,* Δ*sll1951*, Δ*slr2001-slr2002,* Δ*slr1710,* Δ*slr2132*	197 ± 14 mg/L	photoautotrophic; Bubbled with 1% CO_2_	[44]
		Promoter *psbA2, rbc, cpc, trc*			
	Isoprene	*ispS* (codon optimized)	50 μg/g DCW/day	photoautotrophic	[93]
		Promoter *psbA2*			
	Alk(a/e)nes	*sll0208 & sll0209*	2.3 mg/L/OD_730_	photoautotrophic	[94]
		Promoter *rbc*			
	Fatty alcohols	*far, at3g11980* Δ*agp,* Δ*phaAB*	761 ± 216 µg/g DCW	photoautotrophic	[95]
		Promoter *psbD13, rbcL12*			
	Sucrose	*sps, spp,* *ugp* Δ*ggpS*	35 mg/L/OD_730_	photoautotrophic with 600 mM NaCl	[96]
		Promoter *petE*			
	Hydrogen	Δ*narB*, Δ*nirA*	186 nmol/mg chl a/h	nitrogen-limiting in the dark	[97]
***Synechococcus*** **7002**	Hydrogen	Δ*ldhA*	14.1 mol per day per 10^17^ cells	Anaerobic in the dark	[98]
	Sucrose	Δ*glgA-1*, Δ*glgA-II*	71 ± 3 mol per 10^17^ cells	Under hypersaline condition	[99]
***Synechococcus*** **7942**	Ethanol	*pdc* and *adhII*	0.23 g/L	photoautotrophic	[43]
		Promoter *rbcLS*			
	Isobutyraldehyde	*kivd, alsS, ilvC, ilvD* and *rbclS*	1.1 g/L	photoautotrophic with NaHCO_3_	[100]
		Promoter *LlacO_1_,* *trc, tac*			
	Isobutanol	*kivd, alsS, ilvCD* and *yqhD*	0.45 g/L	photoautotrophic with NaHCO_3_	[100]
		Promoter *LlacO_1_, trc*			
	Fatty acids	*tesA* and Δ*aas*	80 ± 10 mg/DCW	photoautotrophic; Bubbled with CO_2_	[101]
		Promoter *trc*			
	Hydrogen	*hydEF, hydG, hydA*	2.8 µmol/h/mg Chl-a	Anaerobic in the dark	[102]
		Promoter *psbA1, lac*			

### 6.1. Biofuels

Several biofuels have been recently biosynthesized in engineered cyanobacteria, including ethanol, butanol, isobutanol, fatty acids, and fatty alcohols [93,95,100,103,104]. Among them, ethanol, isobutanol, fatty acids, and fatty alcohols were produced in *Synechocystis* 6803 [44,92,95,105,106]. *Synechocystis* 6803 has a native transhydrogenase (*slr1434* and *slr1239*, [107]), thus, it is suitable for alcohol production since transhydrogenase can supply NADH from the dephosphorylation of NADPH. Taking isobutanol as an example, Varman* et al.* engineered a *Synechocystis* 6803 strain that can produce isobutanol [105]. In this case, two genes from the *Lactococcus lactis* Ehrlich pathway, *kivD* and *adhA*, which are involved in converting 2-ketoisovalerate into isobutanol, are heterologously expressed in *Synechocystis* 6803 under the control of P_tac_. This strain produced 298 and 114 mg/L of isobutanol under mixotrophic and autotrophic conditions, respectively. For fatty alcohols production in *Synechocystis* 6803, Qi* et al.* constructed a mutant strain by integrating multiple copies of fatty acyl-CoA reductase gene into the chromosome and disrupting the native glycogen/poly-β-hydroxybutyrate biosynthetic pathways (generated 761 µg fatty alcohols/g dry cell weight) [95]. Moreover, fatty acids (precursors for diesel fuels) were produced in* Synechocystis* 6803 after several rounds of genetic modification and optimization, achieving 197 mg/L [44]. Finally, H_2_ is a clean biofuel, which is also widely used for upgrading fossil fuels and for chemical synthesis in the petroleum and chemical industries. *Synechocystis* 6803 can use its oxygen sensitive and bidirectional hydrogenase to synthesize H_2_. Current *Synechocystis* H_2_ production requires an anaerobic atmosphere and the inactivation of photosystems II (such as a dark condition). *Synechocystis* H_2_ evolution can be enhanced by supplying glucose into culture medium [108]. To improve cyanobacterial H_2_ production, redirecting the electron supply (e.g., disrupt nitrate assimilation pathway) and heterologous expression of hydrogenase are two effective strategies of metabolic engineering (Table 1).

### 6.2. Commodity Chemical

A variety of commodity chemicals have been recently biosynthesized in *Synechocystis* 6803. Acetone is a common solvent, and biosynthesis of acetone in *Clostridia* and recombinant *E. coli* has been achieved [109]. Zhou* et al.* have recently designed a novel acetone biosynthetic pathway in *Synechocystis* 6803 to synthesize acetone from CO_2_ [110]. In this pathway, two *C. acetobutylicum* genes encoding an acetoacetate decarboxylase and a coenzyme A transferase are integrated into *Synechocystis* 6803 chromosome [110]. In addition, the native polyhydroxybutyrate (PHB) synthase gene *phaCE* and the phosphotransacetylase encoding gene *pta* were both deleted to reduce the competitive consumption of acetyl-CoA and acetoacetyl-CoA. The acetone titer of the final engineered strain reached 36 mg/L. 

Moreover, ethylene and isoprene are important precursors for the production of synthetic rubbers and polymer. Ethylene has been biosynthesized in *Synechocystis* 6803 by the expression of codon-optimized ethylene-forming-enzyme encoding gene (*efe*) from *Pseudomonas syringae* [111,112]. The ethylene production rate of the engineered *Synechocystis* 6803 can reach to 171 mg/L per day after the optimization of *efe* expression levels, light intensity, and nutrient status [112]. Isoprene was produced in *Synechocystis* 6803 by the expression of a codon optimized isoprene synthase from *Pueraria montana*, which converted the dimethylallyl diphosphate from the native methyl-erythritol-4-phosphate (MEP) pathway to isoprene [93]. In addition, lactic acid is used in the food and pharmaceutical industries [113]. Recently, Angermayr* et al.* have accomplished the photosynthetic production of l-lactate in *Synechocystis* 6803 by integrating the *Bacillus subtilis* *ldh* gene, which encodes an l-lactate dehydrogenase, into the host’s genome [114]. Coexpression of a transhydrogenase led to another 5-fold increase in l-lactate production (up to 288 mg/L), making its titer higher than that from other cyanobacteria, such as *Synechococcus elongates* PCC 7942 (~56 mg/L). 

### 6.3. Polyesters

Biopolymer polyhydroxyalkanoate (PHA) can be biosynthesized in *Synechocystis* 6803 [115]. The PHA biosynthetic operon from *Ralstonia eutropha* was introduced into *Synechocystis* 6803, leading to a two-fold enhancement in PHA synthase activity [116]. (*S*)- and (*R*)-3-hydroxybutyrate (3HB) serves as the building blocks for PHA biosynthesis. An engineered *Synechocystis* 6803 strain (overexpress thioesterase and acetoacetyl-CoA reductases, and knockout polyhydroxybutyrate polymerase) was developed to produce up to 533 mg/L 3HB [117]. Polyhyroxybutyrate (PHB), the most common type of PHA, is naturally accumulated in *Synechocystis* 6803 under nitrogen-starved or phosphorus-starved conditions (granules up to 0.8 micron, 27 mg/L) [118]. Studies by rational gene disruptions have led to high levels of PHB accumulation in *Synechocystis* 6803. Transposon insertion was also used to probe the PHB biosynthetic pathway. It is found that disruption of *sll0461*, a gene encoding the gamma-glutamyl phosphate reductase (*proA*), and *sll0565*, a gene encoding an unknown protein, improved PHB accumulation [119].

In summary, although genetic modification of *Synechocystis* 6803 is more difficult than that of *E. coli*, various products can be successfully produced by engineered *Synechocystis* 6803. 

## 7. Challenges and Perspectives

While large-scale production of value-added products using heterotrophic engineered microbes (e.g., *E. coli* and *S. cerevisiae*) have been demonstrated [120], there are several roadblocks in using autotrophic *Synechocystis* 6803 as a cell factory.

### 7.1. Low Product Titer and Rate

At the current stage, production titers and rates from engineered *Synechocystis* 6803 cannot compete with that from heterotrophic fermentation. For example, fatty acids were produced in an engineered *E. coli* at 5.2 g/L with 73% of the theoretical yield in three days [121]. However, when a similar pathway was introduced to* Synechocystis* 6803, the highest fatty acid titer was below 0.2 g/L after 2-week cultivation [44]. Similarly, when an engineered fatty alcohol pathway containing a thioesterase, an acyl-CoA synthase, and a fatty acyl-CoA reductase was expressed in *E. coli*, fatty alcohols were produced at ~600 mg/L [122]. The titer in *Synechocystis* 6803 from the same pathway was 0.2 mg/L, 3000-fold lower than *E. coli* production [106]. The lower titer and synthesis efficiency may be due to many reasons: lower protein expression level, inefficient NADH/NADPH-generating pathways, and slower cell metabolisms. In general, for most products synthesized by cyanobacterial species, titers are below 1 g/L (Table 1). Moreover, most successfully engineered heterologous pathways are short in cyanobacteria, often containing ~2 to 5 heterologous enzymes. As heterologous pathways become larger and more complicated, metabolic optimizations of cyanobacterial species become increasingly difficult. Therefore, *Synechocystis* 6803 may not be an ideal platform for the biosynthesis of products from complicated heterologous pathways, at least at the current stage. 

### 7.2. Product Loss and Process Failure during Long-Term Cultivation

Cyanobacterial-based biosynthesis is often slower than heterotrophic bacterial fermentation. It often takes cyanobacteria weeks to synthesize a chemical to reasonable titers, increasing the cost for operation and maintenance. Long-period cultivation may increase the risk of microbial contaminations by bacteria or viruses. During this long phototrophic cultivation process, unwanted photo-chemical reactions may occur, leading to product degradation. For example, isobutanol can be slowly lost under light conditions [105]. Furthermore, algal photo-bioreactors often suffer from bio-film fouling during long-term incubation, leading to reduction of light penetration and phototrophic efficiency. Additional efforts for photo-bioreactor cleaning are required. 

### 7.3. Production Costs and Scale up

Commercial large-scale microalgae facilities have been established to produce food supplements, such as β-carotene, astaxanthin, and polyunsaturated fatty acid since 1960s [123]. Previous industrial studies have shown that cyanobacterial bioprocesses face several challenges. For example, large-scale cyanobacterial cultivation can be performed in either open ponds or closed bioreactors [123]. Open pond cyanobacterial cultivation requires adequate sunlight and warm temperatures all year round, therefore placing strong geographic limitations on their production. Open pond cultivation not only has the disadvantage of loss of water due to evaporation, but also has contamination risks by waterfowl, insects, and fungi. More importantly, environmental issues will be raised if genetically modified organisms are used in outdoor ponds. On the other hand, closed bioreactors can be easily controlled (temperature, light and CO_2_ supply conditions) and are less susceptible to contamination [124]. However, they are more expensive to operate. 

Furthermore, producing gaseous products by photosynthetic hosts is challenging due to the use of CO_2_ and product harvesting. For example, Algenol has developed a process to produce ethanol directly from cyanobacteria [125]. A flexible plastic film photobioreactor has to be built to facilitate the product collection. In addition, cyanobacterial processes use large amounts of water, and thus require energy intensive product harvesting processes. Thereby, production of molecules with low margins (such as commodity chemicals and fuels) often needs extensive process design to maximize both yield and productivity. It might be more feasible to couple a cyanobacterial-based bioprocess with the wastewater treatment to improve its economical margins. Under all circumstances, rigorous cradle-to-gate life cycle analysis and economic analysis are necessary to evaluate the applicability and environmental benefits of the cyanobacterial bioprocess. 

## 8. Conclusions

Systems analysis and metabolic modification of *Synechocystis* 6803 are now mainly studied in the laboratory. Although *Synechocystis* 6803 has become a potential photosynthetic host, the productivity of engineered *Synechocystis* 6803 strains are still low as compared to that from *E. coli* and *S. cerevisiae*. In order to take the photosynthetic bio-production to industrial scales, low efficiency of cell metabolisms and limitations in cyanobacterial bioprocesses need to be solved by microbiologists, synthetic biologists, and chemical engineers. Meanwhile, both economic benefits and environmental risks for the use of genetically modified *Synechocystis* 6803 should be rigorously investigated for the cyanobacterial industry. 
